# The Association Between Serum Complement 4 and Kidney Disease Progression in Idiopathic Membranous Nephropathy: A Multicenter Retrospective Cohort Study

**DOI:** 10.3389/fimmu.2022.896654

**Published:** 2022-05-30

**Authors:** Jing Liu, Yang Zha, Peng Zhang, Peng He, Lijie He

**Affiliations:** ^1^ Department of Nephrology, Xijing Hospital, the Fourth Military Medical University, Xi’an, China; ^2^ Department of Postgraduate Student, Xi’an Medical University, Xi’an, China

**Keywords:** serum complement 4, idiopathic membranous nephropathy, renal function progression, risk factor, progression

## Abstract

**Introduction:**

Complement system plays an important role in the pathogenesis of idiopathic membranous nephropathy (IMN), however, the relationship between serum complement 4 (C4) and kidney disease progression in IMN is unclear. This study aims to investigate the association of serum C4 level with the risk of kidney disease progression among patients with IMN.

**Methods:**

The retrospective cohort assessed 1,254 participants with biopsy-proven IMN from three centers in Xi ‘an, Shaanxi Province, China. Baseline serum C4 levels were measured at renal biopsy. The association between baseline serum C4 and the risk of renal function progression, defined as a 30% decline in renal function or end stage renal disease, was evaluated in Cox proportional hazards models.

**Results:**

A total of 328 patients with IMN and nephrotic proteinuria were eligible, and 11.3% (37/328) of them attained the renal function progression events after a median follow-up of 51 months (37-59 months). After adjustment for other confounders, a higher value of serum C4 was independently associated with a higher risk of renal function progression event with a hazard ratio (HR) of 4.76 (95% confidence interval [95% CI], 1.77-12.79) per natural log-transformed C4. In reference to the low level of C4, the adjusted HRs were 2.72 (95% CI, 1.02-7.24) and 3.65 (95% CI, 1.39-9.60), respectively, for the median and high levels of C4 (*P* for trend=0.008). Additionally, the results were robust and reliable in the sensitivity and subgroup analyses.

**Conclusion:**

Among patients with IMN and nephrotic proteinuria, serum C4 at renal biopsy is an independent predictor for kidney disease progression regardless of other confounders.

## Introduction

Idiopathic membranous nephropathy (IMN) is one of the most common forms of primary glomerulonephritis causing nephrotic syndrome in adults, characterized by the formation of immune deposits, complement-mediated proteinuria, and risk of renal failure (1, 2). The differences in the natural course of IMN are significant. Although spontaneous remission occurs in approximately one third of IMN patients, 30-40% of the patients progress toward end-stage renal disease (ESRD) within 5-15 years (3, 4). In particular, approximately 50% of patients with IMN and nephrotic range proteinuria will develop ESRD without treatment (5). Some patients resistant to immunosuppressive therapy will also develop ESRD (6). Therefore, searching for useful markers to predict possible renal outcomes, especially those based on disease mechanisms, is needed and crucial for treatment options in those with IMN.

It has long been known that complement system is activated in immune complex glomerulonephritis to mediate kidney damage (7). Recently, the role of circulatory complement in the development of glomerular diseases has attracted more and more attention. Bi T et al. suggested that serum complement 4 (C4) was independently associated with kidney disease progression in IgA nephropathy (IgAN) with a hazard ratio of 6.98 (95% confidence interval, 1.01 to 48.07, p=0.048) (8). Pan M et al. demonstrated that an increase in serum C4, as well as a decrease in serum C3, was an important determinant of the risk of a >30% decrease in the eGFR for patients with IgAN (9). Additionally, Tsai S et al. indicated that a low serum C3 level predicted poor long-term renal survivals (death or ESRD) in the biopsy-proven IMN (10). The complement system is a cascade of proteins that mediate important innate immune functions but this system’s inappropriate activation has been implicated in kidney disease. Complement activation has been proved to be the central mechanism causing glomerular injury in many forms of glomerulonephritis (11). IMN damages podocyte foot processes due to the fact that the terminal membrane attack complex insertion in podocytes of complement activation causes production of reactive oxygen species, proteases, extracellular matrix, and secretion of transforming growth factor-b, which leads to the loss of slit diaphragm function of podocytes and the leakage of protein from glomeruli (12, 13).

Serum C4, as an active unit of complement cascades, has been measured widely in clinical practice for years. However, its clinical significance remains uncertain for predicting adverse renal outcomes among patients with IMN. Hence, we performed this retrospective cohort study to investigate the association between the level of serum C4 at renal biopsy and the risk of kidney disease progression among patients with IMN.

## Materials and Methods

### Patient Selection

A total of 1,254 participants with biopsy-proven IMN were enrolled from three centers (Departments of Nephrology of the Xijing Hospital, Shaanxi Provincial Hospital of Traditional Chinese Medicine, and Affiliated Hospital of Yan^’^an University) in Xi’an, China. The interval of enrollment was from October 1, 2015 to June 30, 2019. Other inclusion criteria were as follows: (a) estimated glomerular filtration rate (eGFR, calculated using CKD-EPI formula (14)) >15 ml/min/1.73 m^2^, (b) patients with follow-up of ≥ 18 months, (c) patients with nephrotic proteinuria (24-h urinary protein excretion ≥ 3.5g/d), and (d) patients with complete data. The exclusion criteria were as follows: (a) secondary MN, e.g. MN caused by infections (hepatitis B virus, human immunodeficiency virus, syphilis), malignancy (solid tumors [lung, prostate], mesothelioma, some benign tumors), drugs (non-steroidal anti-inflammatory drugs, d-penicillamine, bucillamine), or autoimmune diseases (systemic lupus erythematosus, IgAN, and ANCA-associated vasculitis) et al, (b) biopsy-proven atypical MN, (c) along with other glomerular diseases, e.g. focal segmental glomerular sclerosis, mesangial proliferative glomerulonephritis, and (d) patients with immunosuppressive agents 6 months before renal biopsy.

### Data Collection

All relevant clinical information regarding the eligible patients was retrieved from their medical records, retrospectively. The complete baseline data were collected at renal biopsy, including demographic characteristics (sex, age, body mass index [BMI], smoking status, and blood pressure), laboratory data (serum albumin, anti-PLA2R antibody, C3, C4, immunoglobulin G (IgG), cholesterol, creatinine, and 24-h urinary protein excretion, et al.), and treatment regimens within the first 12 months (renin-angiotensin-aldosterone system [RAAS] blockades, statins, anticoagulations, glucocorticoids, and other immunosuppressive agents). The titers of serum anti-PLA2R antibody were tested using indirect immunofluorescence assays and reported according to the fluorescence intensities and dilutions (1:10, 1:100, 1:1000) of the serum samples. The follow-up data included serum albumin, creatinine, and proteinuria at each visit and was last updated on November 30, 2021.

### Serum C4

Serum C4 was estimated using enzyme-linked immunosorbent assay (ELISA, Uscn Life Science, Wuhan, China). Serum C4 was examined by an independent experienced clinical technician following the manufacturer’s instructions. The steps were as follows: (a) murine monoclonal antibodies binding specifically to the complement components coated on the microarray plates, (b) plasma samples were added according to the optimal dilution, incubation time and room temperature from the manufacturer’s instructions, (c) horseradish peroxidase conjugated antibodies were added and bound to the complement components adsorbed on the plates, and (d) chromogenic substrate was added to ascertain the concentration of C4 (15, 16).

### Definitions and Outcomes

The follow-up time was defined as the interval between renal biopsy and the last outpatient visit, death, or ESRD, whichever occurred first. Body mass index (BMI) was calculated by taking a person’s weight, in kilograms, divided by their height, in meters squared. Hypertension was defined as a systolic pressure of ≥140 mmHg and/or a diastolic pressure of ≥90 mmHg at rest, or use of antihypertension medication. Mean arterial pressure (MAP) was calculated as diastolic blood pressure plus a third of the pulse pressure. A high level of serum anti-PLA2R antibody was defined by a titer of ≥1:100. ESRD was defined by an eGFR value of <15 mL/min/1.73 m^2^ or the initiation of renal replacement therapy.

For IMN, partial remission (PR) was defined by a proteinuria value of ≥0.3 but <3.5 g/24 h plus a 50% reduction from its baseline level at least along with a normal serum albumin (serum albumin ≥3.5 g/dl) and a stable renal function. Complete remission (CR) was defined by a proteinuria value of <0.3 g/24 h, a normal serum albumin, and a stable renal function in at least two consecutive visits. Relapse was defined by the recurrence of proteinuria ≥3.5 g/24 h. No remission (NR) was defined by (1) a proteinuria value of ≥3.5 g/24 h, or (2) <50% decrease in proteinuria from baseline level, or (3) a serum albumin value of <3.5g/dl, or (4) a ≥40% decline in the eGFR prior to achieving proteinuria reduction.

The primary endpoint of this study was renal function progression, defined as a >30% decrease in the eGFR or ESRD. The secondary outcome was defined as a >50% decrease in the eGFR or ESRD.

### Statistical Analysis

Continuous variables with normal distribution were expressed as mean with standard deviation (SD). Otherwise, median with interquartile range (IQR) and the nonparametric test were used. Categorical variables were summarized as frequencies with percentages and compared using χ^2^-test. The levels of baseline serum C4 were expressed as a continuous variable (natural log-transformation) and as a categorical variable (three groups, by tertiles). Spearman’s correlation was applied to analyze the association of serum C4 with clinical parameters including age, BMI, MAP, albumin, complement 3, IgG, cholesterol, serum creatinine, eGFR, microhematuria, and proteinuria.

Kaplan-Meier analyses was used to derive cumulative kidney survival curves and differences between curves were analyzed using a log-rank test. Unadjusted and adjusted Cox proportional hazards regression models were used to analyze the association between serum C4 levels and the risk of a renal function progression event. The magnitude of the relationship was expressed as hazard ratios (HRs) with 95% confidence intervals (95% CIs). Model 1 was adjusted for sex (man or woman), age, and MAP. Model 2 was adjusted for covariates in model 1 plus eGFR, proteinuria, albumin and anti-PLA2R antibody (negative or positive or unknown). Model 3 was adjusted for covariates in model 2 plus the treatment with immunosuppressive agents (none or monotherapy or combination therapy). *P* values for trend were calculated by regarding the rank classified variable of C4 as a continuous variable.

Sensitivity analyses were done by (1) restricting the endpoint to 50% decline in the eGFR or ESRD, and (2) re-analyzing the data of Xijing Hospital. Subgroup analyses were performed by age (<60 and ≥60 years), sex (woman and man), hypertension (yes and no), eGFR (≥90 and <90 ml/min per 1.73 m^2^) and albumin (≥3 and <3 g/dl). The statistical software SPSS version 26.0 (SPSS, Chicago, IL) and GraphPad Prism 7 (GraphPad Software) were used. A two-tailed *P* value of <0.05 was considered statistical significance.

## Results

### Study Cohort

**Figure 1:**
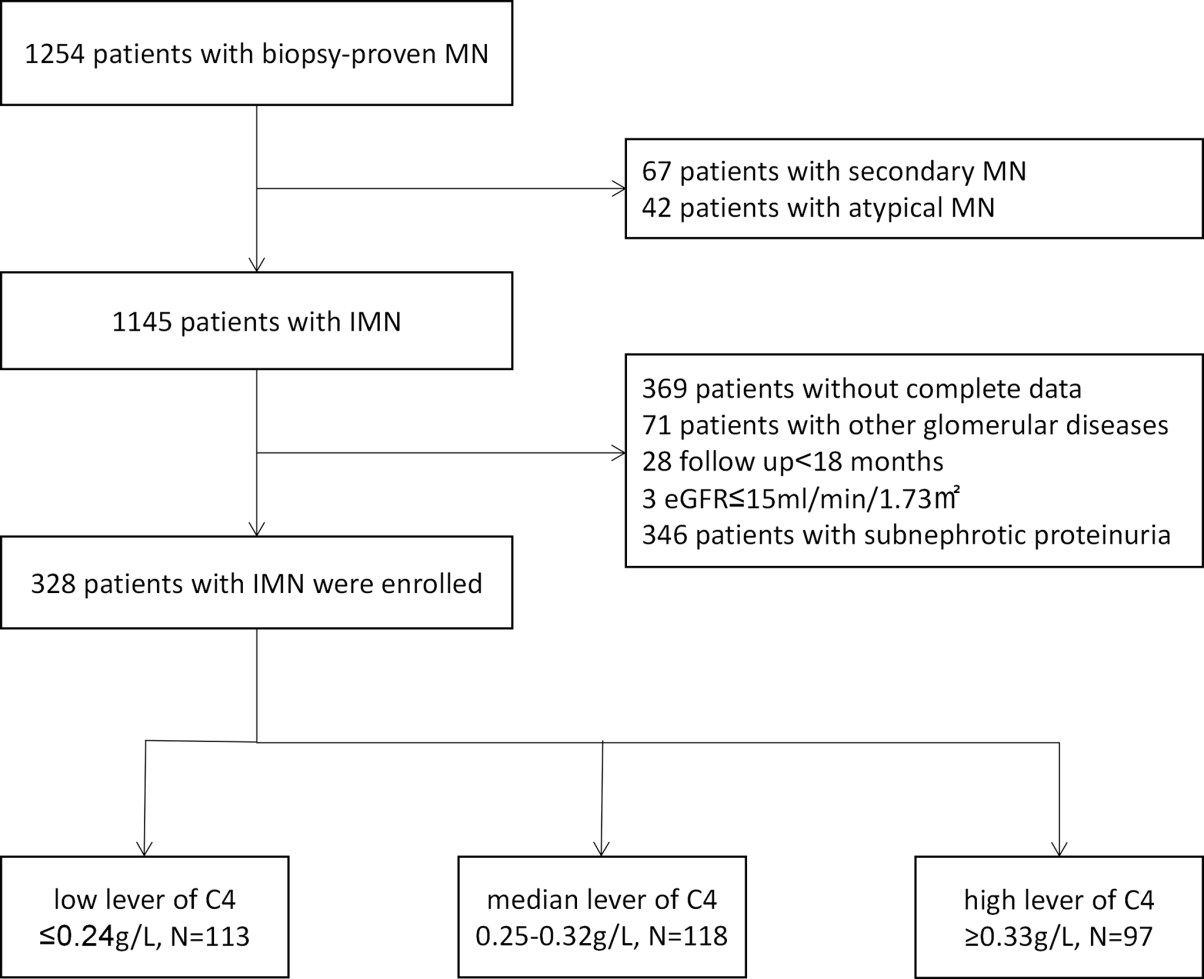
Patient selection flowchart. MN, membranous nephropathy; IMN, idiopathic membranous nephropathy.

Three hundred and twenty-eight patients were included in this study. The flowchart of the patient selection process is displayed in **Figure 1**. The characteristics of the study population and measurements of C4 at the time of kidney biopsy are described in **Table 1**. This cohort included 239 men (72.9%) with the average age of 46.96 ± 14.35 years, the average BMI of 25.41 ± 3.49 kg/m^2^, and the average mean arterial pressure of 94.90 ± 12.59 mmHg. A total of 95 participants (29.0%) were current smokers. At the time of diagnosis, the mean serum albumin, serum complement 3, serum creatinine and eGFR were 2.56 ± 0.57 g/dl, 1.12 ± 0.23 g/L, 0.89 ± 0.27 g/L and 96.52 ± 21.45 ml/min per 1.73 m^2^ (range, 17.13-146.54 ml/min per 1.73 m^2^), respectively. The median serum immunoglobulin G, cholesterol, and proteinuria were 4.98 g/L (IQR, 3.53-6.23 g/L), 294.67 mg/dl (IQR, 234.44-361.85 mg/dl) and 5.89 g/24h (IQR, 4.41-7.98 g/24h). The median microscopic hematuria was 4 RBCs/HPF (IQR, 2.00-9.10 RBCs/HPF), 209 (63.7%) patients a micro- hematuria value of ≥3 RBCs/HPF.

**Table 1:** Characteristics of patients at presentation and subsequent treatments received.

	Total Cohort	Serum complement 4 (g/L, range)			
		Low	Median	High	*p*
		≤0.24	0.25-0.32	≥0.33	
Patient No.	328	113 (34.5)	118 (36.0)	97 (29.6)	0.118
Characteristics at renal biopsy					
Sex (men, %)	239 (72.9)	77 (68.1)	89 (75.4)	73 (75.3)	0.378
Age, yr	46.96 ± 14.35	46.32 ± 12.86	46.73 ± 16.36	48.00 ± 13.43	0.644
BMI, kg/m^2^	25.41 ± 3.49	24.70 ± 3.30	25.67 ± 3.56	25.92 ± 3.52	0.028
Smoke, n (%)	95 (29.0)	35 (31.0)	31 (26.3)	29 (29.9)	0.712
Mean arterial pressure, mmHg	94.90 ± 12.59	93.12 ± 12.29	95.66 ± 13.31	96.06 ± 11.94	0.173
Albumin, g/dl	2.56 ± 0.57	2.63 ± 0.55	2.54 ± 0.55	2.52 ± 0.60	0.290
Complement 3, g/L	1.12 ± 0.23	1.01 ± 0.18	1.12 ± 0.21	1.26 ± 0.24	<0.001
Immunoglobulin G, g/L	4.98 (3.53-6.23)	5.15 (4.04-6.16)	4.69 (3.36-6.39)	5.09 (3.38-6.12)	0.539
Cholesterol, mg/dl	294.67 (234.44-361.85)	288.28 (225.54-330.63)	293.51 (225.74-369.20)	303.56 (254.26-382.74)	0.087
Serum creatinine, mg/dl	0.89 ± 0.27	0.85 ± 0.29	0.94 ± 0.29	0.87 ± 0.21	0.035
eGFR, ml/min per 1.73 m^2^	96.52 ± 21.45	99.71 ± 21.58	93.26 ± 21.67	96.77 ± 20.67	0.072
Microhematuria, RBCs/HPF	4.00 (2.00-9.10)	4.00 (1.84-14.00)	3.00 (1.00-7.00)	5.00 (3.00-9.25)	0.109
Proteinuria, g/24h	5.89 (4.41-7.98)	5.79 (4.15-7.87)	5.89 (4.41-8.11)	6.00 (4.50-7.77)	0.459
High level of serum anti-PLA2R antibody, n (%)	143 (43.6)	52 (46.0)	56 (47.5)	35 (36.1)	0.214
IF-PLA2R staining positivity, n (%)	235 (71.6)	85 (75.2)	84 (71.2)	66 (68.0)	0.772
Post-presentation treatments, n (%)					
RAAS blockades	224 (74.4)	79 (69.9)	91 (77.1)	74 (76.3)	0.400
Statins	245 (74.7)	80 (70.8)	87 (73.7)	78 (80.4)	0.267
Anticoagulant therapy	119 (36.3)	38 (33.6)	41 (34.7)	40 (41.2)	0.474
Immunosuppressive agents					
Monotherapy	36 (11.0)	14 (12.4)	10 (8.5)	12 (12.4)	0.567
Combination therapy	268 (81.7)	90 (79.6)	102 (86.4)	76 (78.4)	

Continuous variables presented as mean ± SD or median (IQR). SD, standard deviation; IQR, interquartile range; BMI, body mass index; eGFR, estimated glomerular filtration rate; RAAS, renin-angiotensin-aldosterone system. BMI was calculated as weight (kg) divided by height (m) squared. Mean arterial pressure was calculated as diastolic blood pressure plus a third of the pulse. eGFR was calculated by CKD-EPI formula.

Also, 143 (43.6%) and 235 (71.6%) patients presented with a high level of serum anti-PLA2R antibody and PLA2R staining positivity.

Overall, 224 patients (74.4%) used RAAS blockades; 245 patients (74.7%) applied statins; 119 (36.3%) patients accepted anticoagulant therapy; 304 participants (92.7%) received immunosuppressive agents (36 [11.0%] monotherapy, and 268 [81.7%] combination therapy).

The median C4 level was 0.28 g/L (IQR, 0.22-0.34 g/L; range, 0.09-1.27 g/L). The participants were next divided into three equal groups (low group/T1, median group/T2, high group/T3) according to the tertiles of C4 distribution (0.25, 0.32 g/L). Among them, 113, 118, and 97 patients occurred in T1, T2 and T3, respectively.

### Outcomes

This study followed up for a median of 51 months (IQR, 37-59 months). After a median follow-up of 7 months (IQR, 4-11 months), 306 (93.3%) participants of the cohort achieved PR. Among those who reached PR, 66 (21.6%) subsequently relapsed in a median of 35 months (IQR, 23-48 months). After a median follow-up of 19 months (IQR, 12-36 months), 206 (62.8%) patients reached CR. Overall, 37 (11.3%) participants reached the renal function progression events. Among them, 37 patients suffered a 30% decline in the renal function and 3 reached ESRD events during follow-up. The median time from kidney biopsy to composite renal endpoint was 48 months (IQR, 35-58 months) (**Table 2**).

**Table 2:** Associations of serum complement 4 with IMN outcomes.

Characteristics	Total Cohort	Serum complement 4 (g/L, range)			
		Low	Median	High	*p*
		≤0.24	0.25-0.32	≥0.33	
Patient No.	328	113	118	97	0.118
Period of follow-up, months	51.00 (37.00-59.00)	48.00 (38.00-58.00)	52.50 (36.75-61.00)	51.00 (37.00-60.50)	0.649
NR, n (%)	22 (6.7)	4 (3.5)	8 (6.8)	10 (10.3)	0.148
PR, n (%)	306 (93.3)	109 (96.5)	110 (93.2)	87 (89.7)	0.148
Relapse, n (%)	66 (21.6)	23 (21.1)	26 (23.6)	17 (19.5)	0.777
CR, n (%)	206 (62.8)	74 (65.5)	71 (60.2)	61 (62.9)	0.705
30% eGFR Decline+ESRD, n (%)	37 (11.3)	6 (5.3)	15 (12.7)	16 (16.5)	0.032
50% eGFR Decline+ESRD, n (%)	16 (4.9)	2 (1.8)	6 (5.1)	8 (8.2)	0.094
ESRD, n (%)	3 (0.9)	1 (0.9)	2 (1.7)	0 (0.0)	0.430
Death, n (%)	7 (2.1)	1 (0.9)	2 (1.7)	4 (4.1)	0.248

NR, no remission; PR, partial remission; CR, complete remission; eGFR, estimated glomerular filtration rate; ESRD, end stage renal disease.

### Correlations Between Serum C4 and Clinical Parameters

As showed in **Table 3**, serum C4 levels showed a positive correlation with BMI (r=0.146, *P*=0.009) and MAP (r=0.127, *P*=0.021). Furthermore, Serum C3 levels (r=0.473, *P*<0.001) and creatinine (r=0.114, *P*=0.039) were demonstrated to have the positive correlation with C4 levels.

**Table 3:** Correlations between serum C4 and clinical parameters.

	Serum Complement 4	
	p	*r*
Age	0.310	0.056
Body mass index	0.009	0.146
Mean arterial pressure	0.021	0.127
Albumin	0.169	-0.076
Complement 3	<0.001	0.473
Immunoglobulin G	0.333	-0.054
Cholesterol	0.079	0.097
Serum creatinine	0.039	0.114
eGFR	0.085	-0.095
Microhematuria	0.911	0.006
Proteinuria	0.745	0.018

eGFR, estimated glomerular filtration rate.

### Association of Serum Complement 4 With Renal Function Progression Event

**Figure 2:**
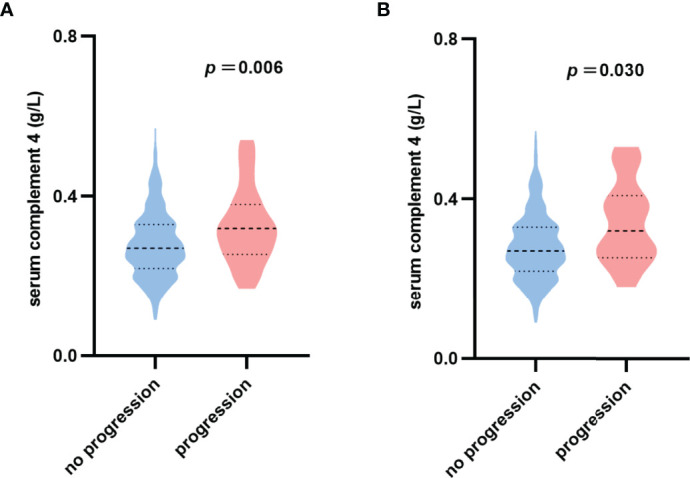
Violin plot of serum complement 4 distribution. **(A)** No progression: patients without reaching 30% decline in eGFR or ESRD; Progression: patients with reaching 30% decline in eGFR or ESRD. **(B)** No progression: patients without reaching 50% decline in eGFR or ESRD; Progression: patients with reaching 50% decline in eGFR or ESRD.

The violin plot of C4 distribution in patients with or without reaching renal function progression events is shown in **Figure 2A**. The median of C4 in patients with renal function progression events was significantly higher in patients without renal function progression events (0.32 versus 0.27 g/L, *p*=0.006). For the endpoint of 50% decline in the eGFR or ESRD, the results were consistent (0.32 versus 0.27 g/L, *p*=0.030) as shown in **Figure 2B**.

**Figure 3:**
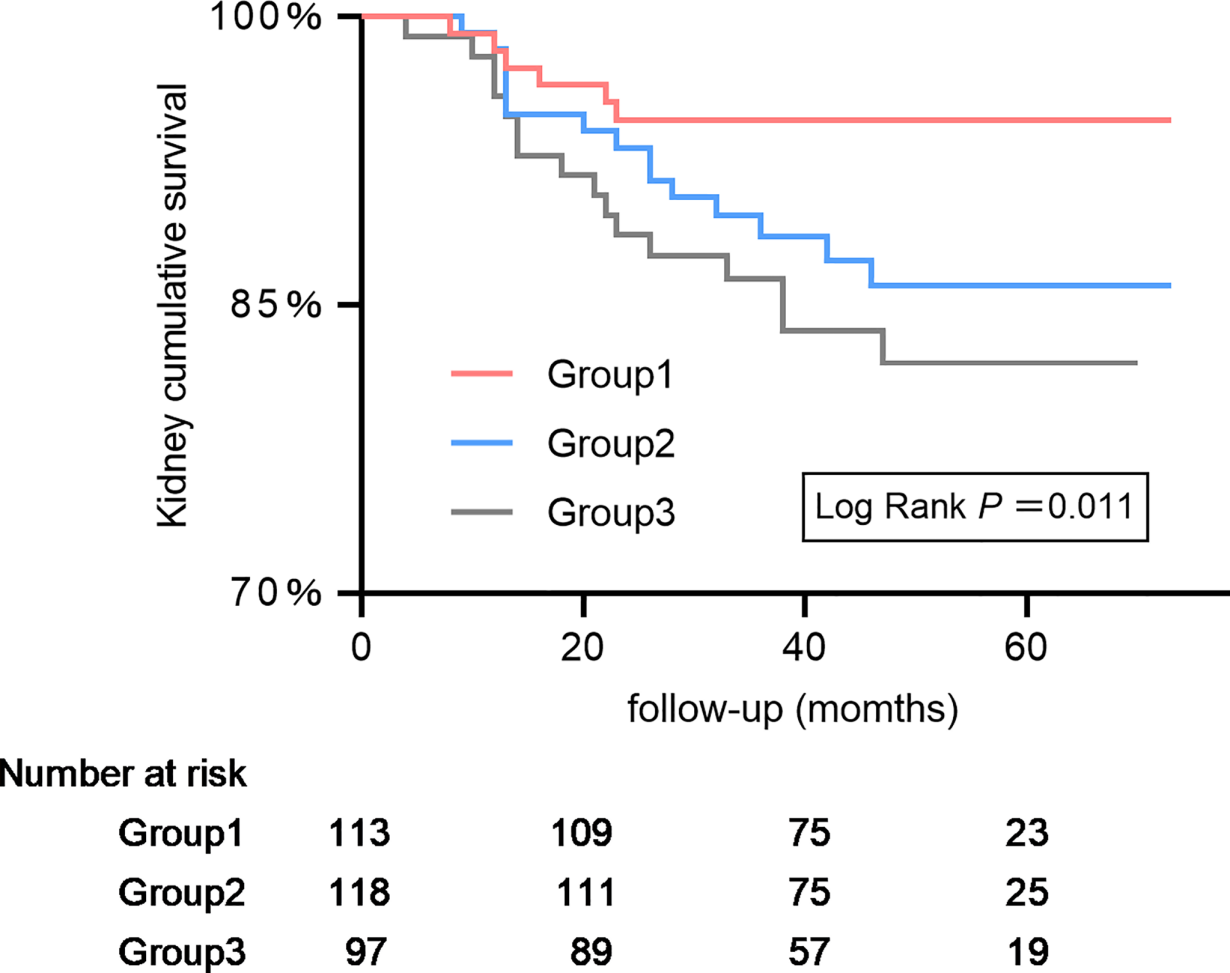
Kaplan–Meier kidney survival curves of participants with idiopathic membranous nephropathy according to serum complement 4. The time zero was kidney biopsy. The division between the three groups of participants was on the basis of tertiles of complement 4.

It depicts the K-M curve of patients with IMN grouped by tertiles (**Figure 3**). The 3-year renal cumulative survival rates of the 3 groups are 94.65%, 88.60% and 86.36%. The 5-year renal cumulative survival rates are 94.65%, 86.00% and 81.99%, respectively. From G1 to G3, the cumulative incidence of renal survival significantly decreased (*P* for trend=0.011).

It shows the Cox proportional hazards progression model of a renal function progression event (**Table 4**). After adjusting for sex, age, MAP, eGFR, proteinuria, albumin, anti-PLA2R antibody and treatment with immunosuppressive agents, higher levels of C4 were independently associated with a greater risk of renal function progression events with a HR of 4.76 (95% CI, 1.77-12.79) per natural log-transformed serum C4. In reference to the low level of C4, the risk of renal function progression events was higher, and the HR was 2.72 (95% CI, 1.02-7.24) for the median level and 3.65 (95% CI, 1.39-9.60) for the high level of C4 (*P* for trend=0.008).

**Table 4:** Cox proportional hazards ratio model of a renal function progression event^a^.

	Hazard Ratio (95% Confidence Interval)			
	Unadjusted model	Model 1	Model 2	Model 3
Serum C4^b^ (per 1 unit greater)	3.52 (1.44-8.61)	3.90 (1.46-10.40)	4.21 (1.60-11.06)	4.76 (1.77-12.79)
Serum C4 tertiles				
1	1.00 (reference)	1.00 (reference)	1.00 (reference)	1.00 (reference)
2	2.43 (0.94-6.26)	2.33 (0.90-6.03)	2.55 (0.96-6.72)	2.72 (1.02-7.24)
3	3.24 (1.27-8.27)	3.10 (1.21-7.97)	3.49 (1.34-9.10)	3.65 (1.39-9.60)
*P* value for trend	0.013	0.018	0.010	0.008

^a^Renal function progression event was defined as a 30% decline in eGFR or ESRD. The events are not mutually exclusive. ^b^Serum C4 was not normally distributed, and the data was converted to normal distribution by natural log transformation. Model 1 was adjusted for sex, age and mean arterial pressure. Sex was analyzed as dichotomous data. Model 2 was adjusted for covariates in model 1 plus eGFR, proteinuria, albumin, and serum anti-PLA2R antibody. Proteinuria was not normally distributed, and the data was converted to normal distribution by natural log transformation. Serum anti-PLA2R antibody was analyzed as dichotomous data (negative or positive or unknown). Model 3 was adjusted for covariates in model 2 plus the treat of immunosuppressive agents (monotherapy or combination therapy or no). Test for trend in Cox regression models were calculated regarding the rank classified variables of serum C4 as continuous variables.

### Sensitivity and Subgroup Analyses

In sensitivity analyses, we recalculated the corresponding effect sizes. In XH center (**Table 5**), after multivariable adjustment, serum C4 at renal biopsy was a risk factor of renal function progression events with a HR of 6.24 (95% CI, 2.12-18.37) per natural log-transformed serum C4. Compared with the low level of C4, the median and high levels of C4 substantially increased the risk of renal function progression events regardless of other confounders. The corresponding adjusted HR values were 4.32 (95% CI, 1.29-14.47) and 6.37 (95% CI, 1.86-21.86), respectively (*P* for trend=0.003). For the endpoint of 50% decline in the eGFR or ESRD (**Table 6**), the corresponding adjusted HR values were 2.81 (95% CI, 0.53-14.93), and 5.33 (95% CI, 1.08-26.38), respectively (*P* for trend=0.028). Additionally, subgroup analyses (shown in **Table 7**) suggests that the association between serum C4 and renal function progression events could not be modified by age (*p* for interaction=0.522), sex (p for interaction=0.294), eGFR (*p* for interaction=0.156), and albumin (*p* for interaction=0.483), regardless of other potential predictors. However, this prognostic relevance was remarkably affected by hypertension (*p* for interaction=0.013). Among episodes of hypertension, a higher C4 level substantially increased the risk of renal function progression events (adjusted HR, 70.62; 95% CI, 3.41-1462.83). Nevertheless, among absences of hypertension, the association between C4 levels and renal function progression events did not appear to be significant (adjusted HR, 2.58; 95% CI, 0.77-8.63).

**Table 5:** Cox proportional hazards ratio model of a renal function progression event^a^ in the Xijing hospital.

	Hazard Ratio (95% Confidence Interval)			
	Unadjusted model	Model 1	Model 2	Model 3
Serum C4^b^ (per 1 unit greater)	3.73 (1.46-9.53)	4.32 (1.53-12.23)	4.53 (1.64-12.58)	6.24 (2.12-18.37)
Serum C4 tertiles				
1	1.00 (reference)	1.00 (reference)	1.00 (reference)	1.00 (reference)
2	3.30 (1.08-10.12)	3.19 (1.03-9.85)	3.51 (1.11-11.15)	4.32 (1.29-14.47)
3	4.21 (1.36-13.04)	4.01 (1.29-12.52)	4.68 (1.47-14.94)	6.37 (1.86-21.86)
*P* value for trend	0.011	0.016	0.008	0.003

^a^Renal function progression event was defined as a 30% decline in eGFR or ESRD. The events are not mutually exclusive. ^b^Serum C4 was not normally distributed, and the data was converted to normal distribution by natural log transformation. Model 1 was adjusted for sex, age and mean arterial pressure. Sex was analyzed as dichotomous data. Model 2 was adjusted for covariates in model 1 plus eGFR, proteinuria, albumin, and serum anti-PLA2R antibody. Proteinuria was not normally distributed, and the data was converted to normal distribution by natural log transformation. Serum anti-PLA2R antibody was analyzed as dichotomous data (negative or positive or unknown). Model 3 was adjusted for covariates in model 2 plus the treat of immunosuppressive agents (monotherapy or combination therapy or no). Test for trend in Cox regression models were calculated regarding the rank classified variables of serum C4 as continuous variables.

**Table 6:** Cox proportional hazards ratio model of a renal function progression event^a^.

	Hazard Ratio (95% Confidence Interval)			
	Unadjusted model	Model 1	Model 2	Model 3
Serum C4^b^ (per 1 unit greater)	4.97 (1.33-18.52)	7.14 (1.42-35.86)	7.09 (1.42-35.47)	8.08 (1.59-41.05)
Serum C4 tertiles				
1	1.00 (reference)	1.00 (reference)	1.00 (reference)	1.00 (reference)
2	2.81 (0.57-13.92)	2.32 (0.46-11.70)	2.59 (0.50-13.43)	2.81 (0.53-14.93)
3	4.86 (1.03-22.91)	4.34 (0.91-20.67)	4.89 (1.01-23.67)	5.33 (1.08-26.38)
*P* value for trend	0.034	0.047	0.035	0.028

^a^Renal function progression event was defined as a 50% decline in eGFR or ESRD. The events are not mutually exclusive. ^b^Serum C4 was not normally distributed, and the data was converted to normal distribution by natural log transformation. Model 1 was adjusted for sex, age and mean arterial pressure. Sex was analyzed as dichotomous data. Model 2 was adjusted for covariates in model 1 plus eGFR, proteinuria, albumin, and serum anti-PLA2R antibody. Proteinuria was not normally distributed, and the data was converted to normal distribution by natural log transformation. Serum anti-PLA2R antibody was analyzed as dichotomous data (negative or positive or unknown). Model 3 was adjusted for covariates in model 2 plus the treat of immunosuppressive agents (monotherapy or combination therapy or no). Test for trend in Cox regression models were calculated regarding the rank classified variables of serum C4 as continuous variables.

**Table 7:** Subgroup analysis of the interactions of renal function progression event with age, sex, hypertension, level of baseline kidney function and baseline albumin using Cox proportional hazard model.

Subgroup	Patient No.	Unadjusted HR (95% Cl)	Model 3	
			Adjusted HR (95% Cl)	*P* for interaction
Age, yr				0.522
<60	264	4.34 (1.49-12.65)	5.87 (1.79-19.29)	
≥60	64	1.63 (0.23-11.77)	1.48 (0.15-15.09)	
Sex				0.294
male	239	4.10 (1.12-15.05)	5.47 (1.37-21.78)	
female	89	3.02 (0.79-11.63)	24.38 (1.07-557.74)	
Hypertension				0.013
No	215	2.05 (0.72-5.85)	2.58 (0.77-8.63)	
Yes	113	69.13 (5.14-930.07)	70.62 (3.41-1462.83)	
eGFR, ml/min per 1.73 m^2^				0.156
≥90	209	2.09 (0.60-7.31)	2.06 (0.45-9.34)	
<90	119	10.00 (1.89-52.87)	12.73 (2.23-72.73)	
Albumin, g/dl				0.483
≥3	77	1.97 (0.47-8.23)	3.84 (0.60-24.51)	
<3	251	5.57 (1.55-20.07)	4.71 (1.19-18.63)	

eGFR, estimated glomerular filtration rate; HR, hazard ratio; CI, confidence interval. Hypertension was defined as diastolic blood pressure >90 mmHg and, or systolic blood pressure > 140 mmHg. eGFR was calculated by CKD-EPI formula.

## Discussion

This multicenter retrospective study enrolled 328 patients with IMN and nephrotic proteinuria and we investigated the association between the level of serum C4 at renal biopsy and kidney disease progression among patients with IMN. Our results showed that a higher level of serum C4 was significantly associated with a higher risk of renal function progression events, which was defined as a 30% decline in the renal function or ESRD, regardless of other confounders.

In renal diseases, it is shown that most glomerular injuries are related to excessive complement activation (17, 18). There was evidence that the level of serum C4 was closely related to the development of chronic kidney disease. In the area of glomerular diseases, especially IgAN, the relationship between serum C4 and long-term kidney function insufficiency has been evaluated (8, 9). However, few studies focused on the association of serum C4 with renal function progression among patients with IMN. In our study, after adjusting for sex, age, MAP, eGFR, serum albumin, serum anti-PLA2R antibody, proteinuria, and treatment with immunosuppressive agents, a higher level of C4 was independently associated with greater risks of renal function progression events with a HR of 4.76 (95% CI, 1.77-12.79) per natural log-transformed serum C4. In reference to the low group, the corresponding HRs were 2.72 (95% CI, 1.02-7.24) and 3.65 (95% CI, 1.39-9.60), respectively, for the median group and high group (*P* for trend=0.008). These results were robust and reliable in our sensitivity and subgroup analyses.

It is very interesting that serum C4 is correlated with renal function progression of patients with IMN. However, the underlying mechanism remains unclear. There are several hypotheses to explain this phenomenon: (1) C4 participates in the classical (antibody-antigen) and lectin (mannan binding lectin [MBL] activation) pathways of the complement system activation. The terminal membrane attack complex of the complement system induces a disturbance of the glomerular filtration barrier (19–23). (2) The split product C4a within the injured kidney is a proximal trigger of many downstream inflammatory events within the renal parenchyma. Aggregation of inflammatory cells release vasoactive substances, causing vascular dilation, increased permeability, leukocyte infiltration, and other inflammatory injuries. Macrophages also have the capacity to synthesize C4. The vicious cycle of C4 and inflammatory cells exacerbate injury to the kidney (7, 24–26). (3) Circulating complement can activate tubular epithelial cells on the vascular lumen surface. These injuring and activating cells produce a microenvironment that promotes fibrillation and inflammation, leading to ESRD (24). (4) There is a progressive increase in the expression of C4 in the tubular epithelial with a subsequent glomerular insult. Local enhancement of C4 synthesis contributes to tissue injury (25, 27).

The search for adequate surrogate markers of kidney disease progression is a key issue in many clinical conditions. For this reason, we speculate that serum C4 has the potential to be a surrogate marker of renal survival for patients with IMN. However, the relationship between serum C4 and hard endpoints needs to be confirmed by prospective and long-term cohorts. The mechanisms of the increase of serum C4 in the progression of IMN should be explored in future research.

The results of our research also suggest that serum C4 levels are correlated with some clinical prognostic factors. Specifically, serum C4 levels are positively correlated with BMI, MAP, C3 and serum creatinine. Yang Y et al. demonstrated that serum C4 was a modest but significantly positive correlation with BMI after adjusted age, gender, cholesterol and triglyceride levels in healthy Hungarian subjects (r=8.645, *p*=0.009) (28). Gaya Da Costa M et al. determined that the level of serum C4 was correlated with C3 (r=0.650, *p*<0.001) in a healthy Caucasian population (29). These were consistent with our conclusions. However, the underlying mechanism is unclear. We surmise that C4 mediates kidney injury, reaction-induced cell apoptosis, detachment of the cells from glomerular basement membrane, degradation of glomerular basement membrane, dislocation of slit diaphragm protein, and inflammatory infiltration resulting in increased creatinine and secondary elevated blood pressure, while increased serum C3 may be due to negative feedback regulation leading the liver to make more of these molecules (4, 30, 31).

One more finding from our study was that the association between C4 levels and renal function progression events was significantly modified by hypertension. At the same time, MAP was positively correlated with serum C4 levers. Bell EK et al. thought that the incidence rate of ESRD was increased at higher levels of MAP in adults with chronic kidney disease (CKD) after multivariable adjustment for socio-demographic and clinical risk factors (adjusted HR,1.54; 95% CI, 1.32-1.79) (32). The kidney disease outcomes quality initiative indicated that hypertension was associated with increased risk for progression of CKD and all-cause mortality in patients with CKD (33). Hypertension and complement are involved in the initiation of IMN, and kidney injury can cause secondary elevated blood pressure that participates in the progression of IMN. This means that serum C4 is more likely to play a predictive role in IMN patients with hypertension. Moreover, these findings also underscore that more attention needs to be paid in the management and prognostic assessment of IMN patients with hypertension. Additional research is warranted to elucidate the relationship between serum C4 and blood pressure of IMN patients and to slow the progression of the disease of these patients.

The strengths of our study include the following: (1) our work is the first study to explore the association of serum C4 and renal function progression among patients with IMN, (2) the multicenter design and sensitivity and subgroup analyses made it possible to validate the robustness of our findings, (3) the advantage of serum C4 lies in the ease of specimen collection, the simplicity of measurement, and the timeliness of report.

The present study also had several limitations: (1) as a retrospective study, we could not avoid its inherent limitations; (2) although we used subgroup analyses to avoid confounding factors, there might be some biases affecting the robustness of our results. At the same time, we could not exclude the potential residual confounders, such as serum anti-PLA2R levels after therapy, and urinary excretion of β2 microglobulin; and (3) we could not have a clear threshold for serum C4, so that the clinical work was limited.

In summary, IMN patients with higher baseline serum C4 levels are at a higher risk for kidney disease progression. Early diagnosis and treatment are important to improve their renal prognosis. However, further prospective studies are needed to verify our findings and the mechanism of this clinical phenomenon remains to be elucidated among patients with IMN.

## Data Availability Statement

The raw data supporting the conclusions of this article will be made available by the authors, without undue reservation.

## Ethics Statement

The studies involving human participants were reviewed and approved by the Institutional Review Board of Xijing Hospital. Written informed consent from the participants’ legal guardian/next of kin was not required to participate in this study in accordance with the national legislation and the institutional requirements.

## Author Contributions

LH, PH, and JL designed the study, analyzed the data, and drafted the manuscript. PH, JL, and YZ collected and entered data. LH, PH, PZ, and JL contributed to the data acquisition and interpretation. All authors read and approved the final manuscript.

## Funding

The study was supported by grants from the National Natural Science Foundation of China (NSFC: 81770764 and 81770669).

## Conflict of Interest

The authors declare that the research was conducted in the absence of any commercial or financial relationships that could be construed as a potential conflict of interest.

## Publisher’s Note

All claims expressed in this article are solely those of the authors and do not necessarily represent those of their affiliated organizations, or those of the publisher, the editors and the reviewers. Any product that may be evaluated in this article, or claim that may be made by its manufacturer, is not guaranteed or endorsed by the publisher.

## References

[1] Ronco P Debiec H. Molecular Pathogenesis of Membranous Nephropathy. Annu Rev Pathol: Mech Dis (2020) 15:287–313. doi: 10.1146/annurev-pathol-020117-043811 31622560

[2] Couser WG. Primary Membranous Nephropathy. Clin J Am Soc Nephro (2017) 12:983–97. doi: 10.2215/CJN.11761116 PMC5460716 28550082

[3] Trujillo H Alonso M Praga M. New Ways of Understanding Membranous Nephropathy. Nephron (2020) 144:261–71. doi: 10.1159/000506948 32229730

[4] Lai WL Yeh TH Chen PM Chan CK Chiang WC Chen YM. Membranous Nephropathy: A Review on the Pathogenesis, Diagnosis, and Treatment. J Formos Med Assoc (2015) 114:102–11. doi: 10.1016/j.jfma.2014.11.002 25558821

[5] Brand AVD Hofstra JM Wetzels JFM. Prognostic Value of Risk Score and Urinary Markers in Idiopathic Membranous Nephropathy. Clin J Am Soc Nephro (2012) 7:1242–8. doi: 10.2215/CJN.00670112 PMC3408119 22595828

[6] Zuo K Wu Y Li SJ Xu F Zeng CH Liu ZH. Long-Term Outcome and Prognostic Factors of Idiopathic Membranous Nephropathy in the Chinese Population. Clin Nephrol (2013) 79:445–53. doi: 10.5414/CN107681 23458172

[7] McCullough JW Renner B Thurman JM. The Role of the Complement System in Acute Kidney Injury. Semin Nephrol (2013) 33:543–56. doi: 10.1016/j.semnephrol.2013.08.005 PMC3816009 24161039

[8] Bi TD Zheng JN Zhang JX Yang LS Liu N Yao L. Serum Complement C4 is an Important Prognostic Factor for IgA Nephropathy: A Retrospective Study. BMC Nephrol (2019) 20:224. doi: 10.1186/s12882-019-1420-0 31272400 PMC6610919

[9] Pan M Zhang J Li Z Jin L Zheng Y Zhou Z. Increased C4 and Decreased C3 Levels are Associated With a Poor Prognosis in Patients With Immunoglobulin A Nephropathy: A Retrospective Study. BMC Nephrol (2017) 18:231. doi: 10.1186/s12882-017-0658-7 28697742 PMC5505039

[10] Tsai S Wu M Chen C. Low Serum C3 Level, High Neutrophil-Lymphocyte-Ratio, and High Platelet-Lymphocyte-Ratio All Predicted Poor Long-Term Renal Survivals in Biopsy-Confirmed Idiopathic Membranous Nephropathy. Sci Rep-Uk (2019) 9:6209. doi: 10.1038/s41598-019-42689-7 PMC6470169 30996263

[11] Kaartinen K Safa A Kotha S Ratti G Meri S. Complement Dysregulation in Glomerulonephritis. Semin Immunol (2019) 45:101–11. doi: 10.1016/j.smim.2019.101331 31711769

[12] Keri KC Blumenthal S Kulkarni V Beck L Chongkrairatanakul T. Primary Membranous Nephropathy: Comprehensive Review and Historical Perspective. Postgrad Med J (2019) 95(1119):23–31. doi: 10.1136/postgradmedj-2018-135729 30683678

[13] Thurman JM. Complement and the Kidney: An Overview. Adv Chronic Kidney Dis (2020) 27(2):86–94. doi: 10.1053/j.ackd.2019.10.003 32553250 PMC7310605

[14] Kong X Ma Y Chen J Luo Q Yu X Li Y. Evaluation of the Chronic Kidney Disease Epidemiology Collaboration Equation for Estimating Glomerular Filtration Rate in the Chinese Population. Nephrol Dial Transpl (2013) 28:641–51. doi: 10.1093/ndt/gfs491 23197682

[15] Zhang MF Cui Z Zhang YM Qu Z Wang X Wang F. Clinical and Prognostic Significance of Glomerular C1q Deposits in Primary MN. Clin Chim Acta (2018) 485:152–7. doi: 10.1016/j.cca.2018.06.050 29969623

[16] Ma R Cui Z Liao YH Zhao MH. Complement Activation Contributes to the Injury and Outcome of Kidney in Human Anti-Glomerular Basement Membrane Disease. J Clin Immunol (2013) 33:172–8. doi: 10.1007/s10875-012-9772-2 22941511

[17] Zhou W Marsh JE Sacks SH. Intrarenal Synthesis of Complement. Kidney Int (2001) 59:1227–35. doi: 10.1046/j.1523-1755.2001.0590041227.x 11260382

[18] Mizuno M Suzuki Y Ito Y. Complement Regulation and Kidney Diseases: Recent Knowledge of the Double-Edged Roles of Complement Activation in Nephrology. Clin Exp Nephrol (2018) 22:3–14. doi: 10.1007/s10157-017-1405-x 28341889

[19] Beck LH Salant DJ. Membranous Nephropathy: Recent Travels and New Roads Ahead. Kidney Int (2010) 77:765–70. doi: 10.1038/ki.2010.34 20182413

[20] Reinhard L Stahl RAK Hoxha E. Is Primary Membranous Nephropathy a Complement Mediated Disease? Mol Immunol (2020) 128:195–204. doi: 10.1016/j.molimm.2020.10.017 33142137

[21] Pippin JW Durvasula R Petermann A Hiromura K Couser WG Shankland SJ. DNA Damage is a Novel Response to Sublytic Complement C5b-9–Induced Injury in Podocytes. J Clin Invest (2003) 111:877–85. doi: 10.1172/JCI200315645 PMC153762 12639994

[22] Yu CY Chung EK Yang Y Blanchong CA Jacobsen N Saxena K. Dancing With Complement C4 and the RP-C4-CYP21-TNX (RCCX) Modules of the Major Histocompatibility Complex. Prog Nucleic Acid Res Mol Biol (2003) 75:217–92. doi: 10.1016/S0079-6603(03)75007-7 14604014

[23] Vignesh P Rawat A Sharma M Singh S. Complement in Autoimmune Diseases. Clin Chim Acta (2017) 465:123–30. doi: 10.1016/j.cca.2016.12.017 28040558

[24] SHEERIN NS SACKS SH. Leaked Protein And Interstitial Damage in the Kidney: Is Complement the Missing Link? Clin Exp Immunol (2002) 130:1–3. doi: 10.1046/j.1365-2249.2002.01979.x PMC1906492 12296845

[25] Welch TR Beischel LS Witte DP. Differential Expression of Complement C3 and C4 in the Human Kidney. J Clin Invest (1993) 92:1451–8. doi: 10.1172/JCI116722 PMC288290 8376597

[26] Carroll MV Sim RB. Complement in Health and Disease. Adv Drug Deliver Rev (2011) 63:965–75. doi: 10.1016/j.addr.2011.06.005 21704094

[27] Zhou W Andrews PA Wang Y Wolff J Pratt J Hartley BR. Evidence for Increased Synthesis of Complement C4 in the Renal Epithelium of Rats With Passive Heymann Nephritis. J Am Soc Nephrol (1997) 8:214–22. doi: 10.1681/ASN.V82214 9048340

[28] Yang Y Chung EK Zhou B Blanchong CA Yu CY Füst G. Diversity in Intrinsic Strengths of the Human Complement System: Serum C4 Protein Concentrations Correlate With C4 Gene Size and Polygenic Variations, Hemolytic Activities, and Body Mass Index. J Immunol (2003) 171:2734–45. doi: 10.4049/jimmunol.171.5.2734 12928427

[29] Gaya Da Costa M Poppelaars F Van Kooten C Mollnes TE Tedesco F Würzner R. Age and Sex-Associated Changes of Complement Activity and Complement Levels in a Healthy Caucasian Population. Front Immunol (2018) 9:2664. doi: 10.3389/fimmu.2018.02664 30515158 PMC6255829

[30] Petermann AT Krofft R Blonski M Hiromura K Vaughn M Pichler R. Podocytes That Detach in Experimental Membranous Nephropathy are Viable. Kidney Int (2003) 64:1222–31. doi: 10.1046/j.1523-1755.2003.00217.x 12969140

[31] Dean MM Minchinton RM Heatley S Eisen DP. Mannose Binding Lectin Acute Phase Activity in Patients With Severe Infection. J Clin Immunol (2005) 25:346–52. doi: 10.1007/s10875-005-4702-1 16133991

[32] Bell EK Gao L Judd S Glasser SP McClellan W Gutiérrez OM. Blood Pressure Indexes and End-Stage Renal Disease Risk in Adults With Chronic Kidney Disease. Am J Hypertens (2012) 25(7):789–96. doi: 10.1038/ajh.2012.48 PMC3784349 22573012

[33] Drawz PE Beddhu S Kramer HJ Rakotz M Rocco MV Whelton PK. Blood Pressure Measurement: A KDOQI Perspective. Am J Kidney Dis (2020) 75(3):426–34. doi: 10.1053/j.ajkd.2019.08.030 PMC7338147 31864820

